# Morphological Formation, Fatty Acid Profile, and Molecular Identification of Some Landraces of Ethiopian Brassica as a Promising Crop to Support Breeding Programs

**DOI:** 10.3390/plants10071431

**Published:** 2021-07-13

**Authors:** Ahmed E. A. Khalaf, Samia A. Abd Al-Aziz, Safaa M. Ali, Adel A. Mohdaly, Mostafa M. Rady, Ali Majrashi, Esmat F. Ali, Ahmed A. M. Yassein

**Affiliations:** 1 Agronomy Department, Faculty of Agriculture, Fayoum University, Fayoum 63514, Egypt; asa14@fayoum.edu.eg; 2 Genetic Engineering and Biotechnology Research Institute (GEBRI), City of Scientific Research and Technology Applications (SRTA-City), New Borg El Arab 21934, Egypt; samiaabdelaziz1@yahoo.com (S.A.A.A.-A.); safaa.mohamedali@yahoo.com (S.M.A.); 3 Food Science and Technology Department, Faculty of Agriculture, Fayoum University, Fayoum 63514, Egypt; aam01@fayoum.edu.eg; 4 Botany Department, Faculty of Agriculture, Fayoum University, Fayoum 63514, Egypt; 5 Department of Biology, College of Science, Taif University, P.O. Box 11099, Taif 21944, Saudi Arabia; aa.majrashi@tu.edu.sa (A.M.); a.esmat@tu.edu.sa (E.F.A.); 6 Genetics Department, Faculty of Agriculture, Fayoum University, Fayoum 63514, Egypt; aay00@fayoum.edu.eg

**Keywords:** *Brassica*, morphological traits, oil quality, internal transcribed spacer, molecular analysis, genetic diversity, path analysis

## Abstract

There has been an increased interest in oilseed crops for agro-industry research and development breeding programs to secure sustainable food and agriculture. The introgression of exotic genotypes of oilseed Brassica into cultivated relatives is inevitable in the genetic improvement of oilseed crops. This experimental attempt aimed to characterize the morphological and molecular basis for the identification and characterization of some Brassica genotypes. Fatty acid profile, yield, and morphology are under genetic control and can be used to identify genotypes. Characterization and identification were fulfilled for five accessions from *Brassica* spp. Plant height, height of first branch, number of branches and pods per plant, seed yield per plant, average pod length, number of seeds per pod, protein and oil contents (%), and fatty acid profile were examined. Besides, the relationship between seed yield and seed yield-contributing characteristics was estimated, as well as the phylogenetic relationship of the internal transcribed spacer (ITS). The genotypes varied significantly for all examined traits, taking into account the most important traits: seed yield per plant and oil content. For example, oil content in the samples ranged between 41.1 and 49.3%. Path analysis results showed a high and positive direct effect between each number of primary branches and the number of pods per plant with seed yield per plant (0.48). The morphological and molecular observations suggest that the Fay1, Fay3, Fay4, and Fay6 accessions belong to *Brassica rapa*, while Fay2 belongs to *Brassica carinata*. It can be concluded based on the present findings that the Fay3 genotype with the highest oil content and the lowest erucic acid content compared to the other genotypes can be proposed as a potential donor for future breeding programs for oil production and quality, while Fay1 can be utilized as donor to increase the seed yield per plant.

## 1. Introduction

The agricultural sector is relied on all over the world to bridge the gap between the steady increase in population and agricultural outputs in order to secure food and sustainable agriculture globally. To achieve this, new promising food crops must be introduced into breeding programs to adapt them for cultivation in the different agricultural areas, each according to its environmental conditions.

The family of Brassicaceae was characterized by morphological and phytochemical traits and had a wide variation. These characteristics have been used to evaluate the diversity between different plant species and to identify cultivars [1,2,3]. The profile of morphological traits is considered under genetic and environmental control. Accurate identification is key to breeding studies and is necessary for several disciplines [4]. To ensure the success of a breeding program aimed at improving desirable traits, it is important to search for wide genetic variance between and/or within species to select valuable parents. Ethiopian mustard is usually cultivated for its oil, which is rich in erucic (~40%) and linoleic acids [5,6,7]. One of the best ways to breed a *Brassica* species is to look at the oil content [8]. A high level of variability is revealed in *B. carinata* [9] and *B. juncea* [10] for some agro-morphological traits. Different tools are available to study genetic variability and the relationships among accessions including morphological characterization, seed yield components, multivariate statistical analyzes, principal component analysis, and molecular markers [11,12]. Different techniques of DNA polymorphism analysis can be used to identify the phylogenetic relationships of plant species. Among these techniques, ITS is a widely used technique in phylogenetic studies of plants [13,14]. ITS sequences have been developed to be distinct for growing species and can be used as a barcode to distinguish between plant species [15,16]. A large amount of variability has been identified in ITS, including the presence of polymorphic copies of ITS [17,18]. Morphological, biochemical, and posterior molecular classification can be used to analyze and identify diversity. Morphological markers have been used mainly for diversity analysis, and are still in practice. Of course, these were naturally occurring variants of a given plant genus. Upon the advancement of genomic tools, molecular markers have become the implemented alternative for evaluating genetic diversity [19,20]. Path analysis may provide insight into a complex relationship between different characteristics in a biological system, as well as information about whether the observed correlation results from direct influence or through other variables. Path Coefficient Analysis is a very important statistical tool that can be successfully used to divide the correlation coefficient into direct and indirect influence of independent variables on the dependent variable, such as yield in rapeseed [21].

The current study aimed to identify promising Ethiopian *Brassica* landraces by using morphological formation, fatty acid profile, and molecular characterization. Given these considerations, the current research also aimed to explore the correlation coefficients among various vigor and vigor contributing traits, as well as to evaluate the direct and indirect effects of vigor on other traits.

## 2. Materials and Methods

### 2.1. Plant Material

After identification, the authors of this study renamed five Ethiopian *Brassica* landraces genotypes as Fay1, Fay2, Fay3, Fay4, and Fay6. These landraces were provided by Dr. Behailo Guta, ONG, Addis Ababa, Ethiopia for use as plant material in this study. The current study was carried out at Demo Farm, which is considered a newly reclaimed soil (Southeast Fayoum; 29°17′ N; 30°53′ E), Fayoum, Egypt, during two successive seasons: 2017/2018 and 2018/2019. A complete randomized block design was used, with three plots as three replications in the two seasons. The experimental area was divided into plots and each plot consisted of 3.5 m ridges 60 cm apart. Planting was conducted in hills that were 10 cm apart on one side of the ridge. Thinning was performed four weeks after planting, and two plants have remained on each hill.

### 2.2. Vegetative Growth Characteristics

Morphological parameters were collected at the harvest stage and measured using 10 random plants in each plot. Observations were recorded on nine traits, namely plant height (PH, cm), height of first branch (HFB, cm), number of primary branches per plant (NPB), number of pods per plant (NPP), average pod length (PL, cm), number of seeds per pod (NSP), seed yield per plant (SYP, g), while seed protein was measured by Near Infrared Analyzer [22].

### 2.3. Extraction of Crude Oil from Seeds

Seeds of *Brassica* spp. were carefully washed to remove any foreign matter, dried to the necessary moisture level, and then crushed by a blender. A conventional method of oil extraction was used [23]. The collected oil was dried over anhydrous sodium sulfate, the mass of oil was determined using the gravimetric method, and the oil was then stored in dark brown bottles at −20 °C until examination. The experimental assays were conducted in triplicate and were completely randomized.

### 2.4. Gas Chromatography Analysis of Oils

After preliminary derivatization to form fatty acid methyl esters (FAME), the fatty acid profiles of the crude oil samples were evaluated using gas chromatography (GC-type CG-2010 Plus, Shimadzu), in agreement with the method described by Mohdaly et al. [24]. The fatty acid composition values were expressed as a percentage (percentage, *w*/*w*).

### 2.5. Morphological Data Analysis

Data were tested for normal distribution. Randomized complete blocks design with three replications was practiced. The resulting data were submitted to analysis of variance (ANOVA) using IBM^®^ SPSS^®^ (SPSS Inc., IBM Corporation, NY, USA) Statistics version 25 for Windows (R). Differences among genotypes were tested with the Bonferroni adjustment correction post-hoc test (Level of significance *p* < 0.05, 0.01, and 0.001) [25].

### 2.6. Path Analysis

In addition to using the correlation coefficient, positive or negative effects for path analysis results were used. Path analysis results are important when direct and indirect effects are interpreted. The importance of the path analysis results lies in the case of interpretation of direct and indirect effects.

Analysis of path coefficients was performed following the procedure developed by Dewey and Lu [26] using IBM SPSS AMOS 24 [27]. Path coefficient analysis was performed assuming that seed yield per plant and oil content (%) functioned as dependent variables, and the other traits were considered as independent variables.

### 2.7. Molecular Characterization

Following the Cetyl Trimethyl Ammonium Bromide (CTAB) extraction procedure, total DNA was extracted from fresh leaves (3–4 weeks old seedling) of plants selected randomly from each genotype [28]. Agarose gel electrophoresis was used to assess the quality and quantity of DNA samples, which were used for setup PCR amplification.

The ITS region of the nuclear rDNA was amplified by PCR using the primers of ITS1 and ITS4 [29]. Clear and sharp amplification bands were purified and sequenced for phylogenetic studies. The amplified fragment sequence 750 bp was identified and analyzed using a 3130 genetic analyzer. Phylogenetic tools have allowed many genera and species to be grouped using traditional taxonomic methods. The sequence was sent to the BLAST database to look for homologies with other ITS sequences. After comparing the sequence of the studied strain with the sequences sent to GeneBank, the percentages of similarity and accession numbers were obtained. Nucleotide variation of the nuclear ITS regions was used to study phylogenetic relationships among the examined genotypes. The fraction of nitrogenous bases in a DNA or RNA molecule that are either guanine (G) or cytosine (C) is known as GC-content (or guanine–cytosine content) (C). This metric represents the percentage of G and C bases in a total of four bases, which includes adenine and thymine in DNA and adenine and uracil in RNA. The Mol (%) of nucleotide A, C, G, and T were calculated using BioEdit 7.2 program to differentiate between nucleotides through selected landraces. Analyses of the ITS gene using different restriction enzymes (*Eco RI, Nco I, Bam HI, Tfi I, AgsI, Eco 571*, and *Cfr10I*) were fulfilled. PCR-Restriction Fragment Length Polymorphism (PCR-RFLP): the ITS PCR products of the five samples were digested using different restriction enzymes. The digested products were fractionated and the *Brassiceae* isolates were characterized.

## 3. Results

### 3.1. Agromorphological Traits

Variance analysis for all agronomic traits as well as the protein content (%) indicates significant differences between accessions over two seasons (Table 1). The Fay1 accession awarded the highest values of plant height, first branch height, number of branches per plant, number of pods per plant, seed yield per plant, protein content (%), average pod length, and number of seeds per pod.

### 3.2. Total Oil Content of Rapeseeds Genotypes

The results indicated that total oil content (%) of the five brassica landraces ranged between 41.1 and 49.3%, with the maximum value in the Fay3 genotype, whereas the Fay1 genotype presented the lowest value of oil content (Table 1).

### 3.3. Fatty Acid Composition

The compositions of fatty acids of different oilseed genotypes were determined through GC analysis, and the results obtained are given in Table 2. In the samples, sixteen different fatty acids were identified and quantified. Erucic, oleic, linoleic, α-linolenic, and paullinic acids were the principal fatty acids identified in oil samples. Erucic acid (C22: 1) was the most abundant fatty acid in most of the rapeseed genotype oil profile; it is toxic and not essential for human growth, and renders the oil unfit for human consumption. Fay1, Fay2, Fay4, and Fay6 genotypes had almost identical abundances of erucic acid at 45.87%, 47.78%, 45.88%, and 44.25%, respectively. On the contrary, the Fay3 genotype showed low content of erucic acid (20.83%) and had the highest content of oleic acid (22.98%). Considering the fact that oleic acid plays a significant role in increasing the nutritional quality of brassica oil, the high oleic acid content of the sample Fay3 is considered very advantageous for health as this fatty acid is suitable for hypo-cholesterol diets, particularly in the preparation of frozen food, and its high stability makes it suitable for use in cooking and frying oils [30]. Our results indicate that breeding efforts to decrease erucic acid content and increase oleic acid content in rapeseed might be successful with the Fay3 genotype. Saturation of single-chain fatty acids, such as stearic acid (18:0), myristic acid (14:0), margaric acid (17:0), arachidic acid (20:0), palmitic acid (16:0), and behenic acid (22:0), takes place. Saturated fatty acids (SFAs) contribute to an increased risk of cardiovascular disease, while monounsaturated fatty acids (MUFAs) and polyunsaturated fatty acids (PUFAs), in the opposite direction, reduce the risk of coronary heart disease (CHD). It was observed that our brassica oil samples had very low levels of SFA (5.1–5.7%) and substantial amounts of MUFA (55.88–62%) and PUFA (31.54–36.91%). Due to their ability to reduce serum cholesterol and Low-Density Lipoprotein (LDL) levels, these genotypes may be used as good sources of essential fatty acids due to their high percentage of linoleic acid [31].

In the samples under review, the amount (%) of unsaturated fatty acids was 92% and above. The proportions of saturated fatty acids to unsaturated fatty acids (SFA/UnSFA), criteria commonly used to describe the nutritional value of an oil, were low for all oil samples (around 0.06%). Based on the analyses, we can conclude that the Fay3 genotype oil can be considered as a greater potential healthy dietary source than other genotypes because it has a low amount of erucic acid and a high proportion of oleic acid. In addition, it is recommended that reductions in erucic acid content from 20% to <2% be achieved in Fay3 genotype oil via further breeding programs.

### 3.4. Path Analysis

**Figure 1:**
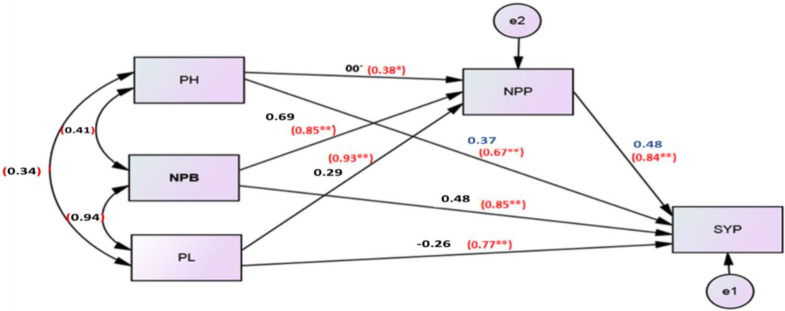
The path analysis for direct and indirect effects and Pearson correlation (r) of plant height (PH), number of primary branches (NPB), pods length (PL) and number of pods/plant (NPP) on seed yield/plant (SYP). ‘*’ means significant at *p* ≤ 0.05 and ‘**’ means significant at *p* ≤ 0.01.

The path analysis approach was used to assess the relationships between seed yield per plant (SYP) as the dependent variable and plant height (PH), number of primary branches per plant (NPB), average pod length (PL), and number of pods per plant (NPP) as independent variables. Relationships between the dependent and independent variables are shown in Figure 1.

The positive direct effects between plant height (PH), number of primary branches per plant (NPB), number of pods per plant (NPP) with seed yield per plant (SYP), respectively, were 0.37, 0.48, and 0.48, whereas the negative direct coefficient was −0.26 for average pod length (PL). NPB and NPP have had the highest direct effect on SYP, while the lowest direct effect was for PH. Likewise, the NPB and PL showed indirect positive effects on SYP through the NPP.

Figure 1 shows the Pearson correlation between SYP and its components. SYP was significantly positive and closely correlated with NPB (0.85), NPP (0.84) and PL (0.77). PH showed a moderate and significant positive correlation with SYP (0.67) and a weak significant positive correlation with NPP (0.38). The direct and indirect effects, Pearson correlation, and coefficient of determination (R^2^) of different agronomic and quality characteristics on the oil percentages of five landraces of *Brassica* are shown in Table 3. The resulting data indicated that, in general, all traits had a negative direct effect on oil content except protein content (0.23). Height to first branch (HFB) contributed the highest positive total indirect effects (2.73) on oil content, while number of pods per plant (NPP) showed the highest negative total indirect effect on oil percent (−0.64). Table 3 also shows the correlation matrix between oil percent and eight traits. Oil content had a strong and statistically significant positive correlation with height to first branches (HFB) (0.77). While oil content had a strong and significant negative correlation with number of pods per plant (NPP) (−0.82), number of primary branches (NPB) (−0.79), number of seeds per pod (−0.78), seed yield per plant (SYP) (−0.76) and average pod length (PL) (−0.74), a weak and significant negative correlation was observed between oil percent and each of the plant height (−0.46) and the protein percent (−0.37).

### 3.5. Molecular Characterization

**Figure 2:**
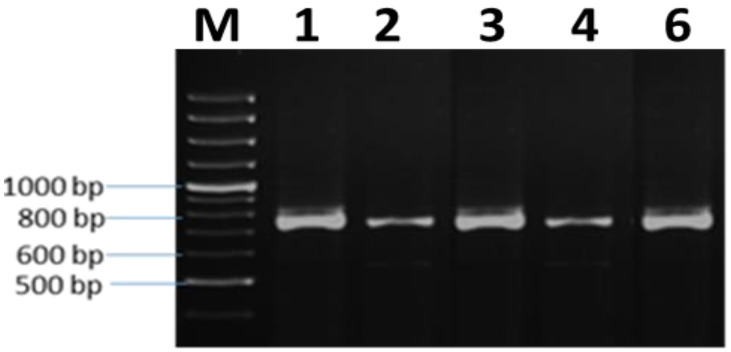
Agarose gel electrophoresis of the amplified PCR fragment for the ITS gene. M: 1 kb DNA marker, isolates with code no. (1, 2, 3, 4 and 6).

DNA was successfully extracted for the five selected accessions. Molecular identification of the selected accessions was identified using PCR-amplified ITS genes. The products of the PCR were analyzed using 1% agarose gel (Figure 2).

**Figure 3:**
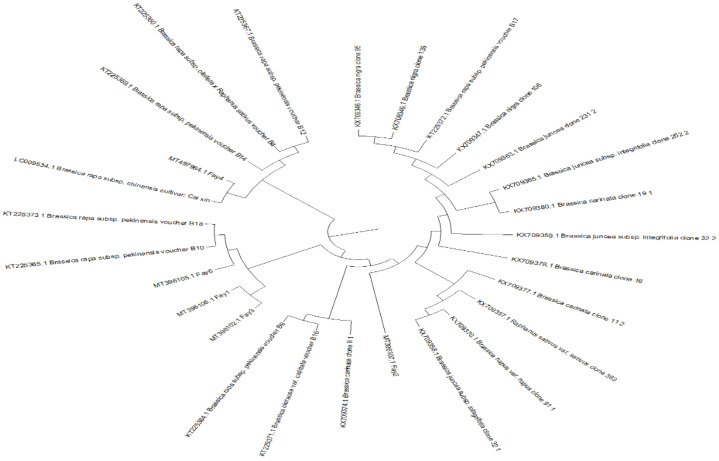
Phylogenetic tree of isolates coded 1, 2, 3, 4 and 6 achieved display the location amid the selected *Brassica* spp. based on ITS sequence assessments.

**Figure 4:**
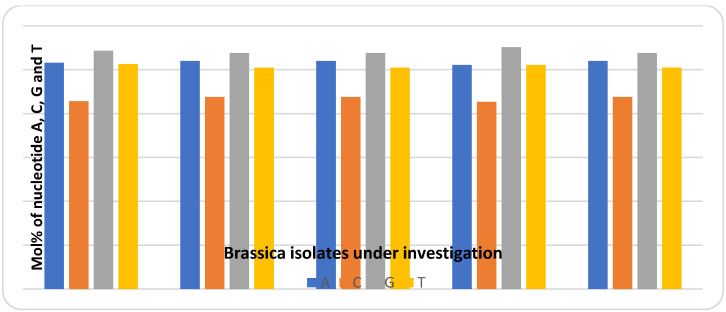
Molecular weight % of nucleotide, Adenin (A), Cytosin (C), Guanin (G) and Thymin (T) in ITS sequences of the *Brassica* accessions under investigation.

Multiple sequence alignments of the ITS regions were conducted to identify individual ITS types, with the others presented in NCBI (Figure 3). The accession numbers in GeneBank for the selected plant accessions were MT396106.1 for Fay1, MT396107.1 for Fay2, MT396102.1 for Fay3, MT497984.1 for Fay4, and MT396105.1 for Fay6. Mega X software was used to construct a phylogenetic tree (Figure 3), which revealed that Fay1, Fay2, Fay3, and Fay4 were more closely related to *B. rapa*, while Fay6 had individual branches in the tree that were more closely related to *B. carinata*. These observations indicated a variety of origins. Guanine–Cytosine (GC) material was found to be very similar in ITS sequences. The GC-content of a DNA or RNA fragment or a whole genome may be determined. It can refer to the GC content of a specific gene or segment of a gene (domain), a collection of genes or gene clusters, a non-coding area, or a synthetic oligonucleotide, such as a primer, when referring to a fragment. The GC content in all accessions was found to be around 49% using the ITS sequence in the current study. The G + C contents (%) of the five Brassica isolates were as follows: Fay3 (48.78), Fay6 (48.78), Fay1 (48.56), Fay2 (48.78), and Fay 4 (48.91). The Mol (%) of nucleotides A, C, G, and T, and the differentiation between nucleotides through selected isolates and data, are presented in Figure 4.

**Figure 5:**
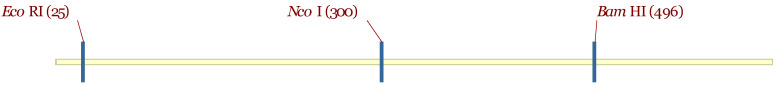
Restriction map of *Eco RI*, *Nco I* and *Bam HI* on the ITS PCR products for Brassica accessions under investigation (Fay1, 2, 3, 4 and 6).

**Figure 6:**
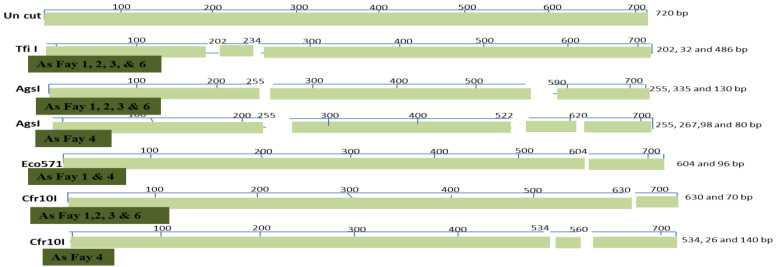
Restriction map of *Tfi I, AgsI, Eco 571*, and *Cfr10I* on the ITS PCR products for Brassica accessions.

All Brassica isolates under investigation contain a single restriction site for *Eco RI*, *Nco I*, and *Bam HI*, as presented in Figure 5; these enzymes have the same restriction site in the five Brassica isolates. On the other hand, *Tfi I*, *AgsI*, *Eco 571*, and *Cfr10I* have a different restriction site, as displayed in the restriction map (Figure 6).

## 4. Discussion

Morphological traits are functioned to evaluate the relationships between yield, oil content, and attributing traits in oilseed plants, as well as to describe and identify promising landraces based on those observations [2,3]. In this regard, in different reports, significant variations of agronomic traits and seed yield in *Carthamus tinctorius* and *B. carinata*, respectively, were noted [32,33,34]. Tiwari et al. [10] separated varieties of *B. juncea* based on morphological characterization, indicating the variability between varieties.

*Brassica* accession oil has a relatively high content of erucic acid, which limits the use of oil for human and animal consumption as it can cause heart damage. Fatty acid profile analysis is very important in nutritional information and helps to understand the availability of various fatty acids between edible oils and food commodities. Fatty acids are the primary component of lipids and can exist as free and bound forms. They can be categorized by hydrocarbon chain into long-chain fatty acids (C13–C21), very long-chain fatty acids (>C22), medium-chain fatty acids (C6–C12), and short-chain fatty acids (<C6). GC is among the most effective methods available in a variety of oil and food products for analyzing the total fatty acids. Oil, which is the most important component of oilseeds and their major source of economic return, is primarily associated with seed oil content and is considered one of the most important factors affecting the success of rapeseed breeding programs [35]. Higher oil content would make the crop more profitable in the context of any proposed end-use. To meet the high demand for oil for large-scale industrial production, increasing oil content or output in rapeseeds is required. Seed oil content in rapeseeds has thus been examined via various genotypes. These findings are consistent with those of [36] who found that the oil contents (%) of different varieties of rapeseed oil from Roma, Italy ranged from 25% to 41%, with an average value of 33.1%, which are exaggerated by environmental surroundings and genotypes. In another study, it was reported that the total oil contents of rapeseed cultivars ranged from 21.0% to 47.3% [37,38].

The higher polyunsaturated fatty acid ratios (PUFA) to SFA (also known as the Polyene index) indicate a more marked susceptibility to autoxidation through fatty acid composition. According to [39,40], UFA has a more favorable impact and effect, and provides greater health benefits, than SFA. The ratio of omega-6/omega-3 of the oil genotypes varied between 1.09 and 1.58 depending on their fatty acid composition. In terms of the presence and number of double bonds in the carbon chain, fatty acids can be divided into four categories: saturated, monounsaturated, polyunsaturated, and trans fats. Kumar et al. [40] reported that erucic acid content (C22:1) ranged from 40.7% to 42.9% over the entire seed base. The linoleic (omega-6) and α-linolenic (omega-3) fatty acids appear to be the most important because they have many beneficial characteristics, such as possessing anti-inflammatory properties, reducing oxidative stress, and presenting neuroprotection and cardiovascular protection; however, there is no definite biochemical pathway for the body to produce these molecules on its own, and thus, they must be obtained from food [41]. The World Health Organization (WHO) estimated that CHD causes 500,000 premature deaths worldwide per year [30].

Overall, our findings are in agreement with those presented in [42], where yield is directly and strongly associated with the number of seeds (0.93 and 0.97), which was related to the number of pods and seeds per pod; selection for these traits may be effective to improve the yield. Despite the exception of protein content (0.23), all traits had a negative direct effect on oil percent. According to [43], similar findings were observed insofar as the day to maturity and the seed yield harmed the oil content; the remainder (0.2311) indicates that characteristics used in the genotypic path analysis clarified 26.99% of the overall oil content variance. A significantly low and negative correlation was observed between oil content and both plant height (−0.46) and protein percent (−0.37). It has been found in [42,44] that *Brassica* seeds trade in terms of oil and protein.

The use of phylogenetic tools has permitted many genera and species to be grouped according to conventional taxonomic techniques. A phylogenetic tree was created using the Mega X program and the findings suggested that the origin was diverse [20,45,46]. Sequences from a gene bank of different species were a dominant method of classification [47]. ITS sequences are widely applied in different field crop plants to study the phylogenetic relationship between plant genera [17,18,48]. In the present findings, using the ITS sequence, the GC content was about 49% in all accessions. ITS sequences showed high similarity to the Guanine–Cytosine (GC) content. The results were congruent with those in [48], which identified and characterized eight species of *Cuscuta* genes based on molecular (ITS) and morphological data and used the analysis of phylogeny to confirm the diversity among *Cuscuta* species. Information about genetic distance among the accessions is helpful in a breeding program [49,50].

## 5. Conclusions

The molecular observations suggest that the Fay1, Fay3, Fay4, and Fay6 accessions are related to *Brassica rapa*, while Fay2 is related to *B. carinata*. Based on the current findings, genotype Fay3, which contains higher oil content and lower erucic acid content than other genotypes, may be used as the source for future oil production and quality breeding programs. The Fay1 accession can be used as a seed donor to increase seed yield per plant. The study’s results can be used to focus potential breeding programs on the genetic improvement of *Brassica*.

## Figures and Tables

**Table 1:** Plant height (cm), height of first branch (cm), number of branches and pods per plant, seed yield per plant (g), length of pods (cm), and number of seeds per pod, as well as protein and oil contents (%) of the evaluation Brassica accessions for two growing seasons.

**Accession**	**Fay1**	**Fay2**	**Fay3**	**Fay4**	**Fay6**
	2017/2018				
Plant height	175.3 ^a^ ± 4.9	145.9 ^b^ ± 4.1	141.8 ^b^ ± 5.1	141.2 ^b^ ± 5.8	142.0 ^b^ ± 1.3
Height of first branch	14.0 ^e^ ± 0.4	32.9 ^b^ ± 0.5	43.5 ^a^ ± 0.8	17.3 ^d^ ± 0.7	23.3 ^c^ ± 0.7
No. of branches per plant	22.2 ^a^ ± 0.8	13.7 ^c^ ± 0.6	8.5 ^d^ ± 0.4	20.7 ^a^ ± 0.5	17.8 ^b^ ± 0.4
No. of pods per plant	2968.7 ^a^ ± 5.3	1992.9 ^d^ ± 3.4	1147.8 ^e^ ± 13.3	2764.3 ^b^ ± 9.4	2536.5 ^c^ ± 18.2
Seed yield per plant	87.8 ^a^ ± 2.6	49.6 ^d^ ± 0.4	37.9 ^e^ ± 0.6	60.2 ^b^ ± 2.22	56.6 ^c^ ± 0.5
Average pod length	5.2 ^a^ ± 0.05	3.3 ^c^ ± 0.1	2.5 ^d^ ± 0.2	5.1 ^a^ ± 0.1	4.7 ^b^ ± 0.1
Number of seeds per pod	21.3 ^a^ ± 0.3	13.0 ^d^ ± 0.7	7.8 ^e^ ± 0.3	19.8 ^b^ ± 0.5	17.3 ^c^ ± 0.3
Protein content	31.2 ^a^ ± 0.2	23.0 ^c^ ± 0.5	26.3 ^b^ ± 1.4	26.8 ^b^ ± 0.2	26.5 ^b^ ± 0.7
Oil content	41.2 ^c^ ± 0.4	44.8 ^b^ ± 0.2	49.2 ^a^ ± 0.5	44.8 ^b^ ± 0.9	42.4 ^c^ ± 0.5
	2018/2019				
Plant height	165.2 ^a^ ± 4.3	160.0 ^a^ ± 2.1	141.0 ^b^ ± 5.1	136.5 ^b^ ± 5.8	134.3 ^b^ ± 5.1
Height of first branch	14.3 ^e^ ± 0.3	33.1 ^b^ ± 0.6	43.8 ^a^ ± 0.9	19.3 ^d^ ± 0.5	23.4 ^c^ ± 0.4
No. of branches per plant	21.8 ^a^ ± 0.6	15.9 ^c^ ± 0.5	7.3 ^d^ ± 0.4	20.3 ^b^ ± 0.5	19.7 ^b^ ± 0.5
No. of pods per plant	2970.3 ^a^ ± 5.6	1979.5 ^d^ ± 3.7	1130.7 ^e^ ± 7.3	2741.3 ^b^ ± 14.2	2565.4 ^c^ ± 11.4
Seed yield per plant	90.3 ^a^ ± 1.1	51.2 ^c^ ± 1.1	37.0 ^d^ ± 0.7	58.2 ^b^ ± 0.8	55.3 ^b^ ± 0.1
Average pod length	5.3 ^a^ ± 0.1	3.7 ^d^ ± 0.1	2.7 ^e^ ± 0.1	4.6 ^b^ ± 0.1	4.1 ^c^ ± 0.1
Number of seeds per pod	20.8 ^a^ ± 0.3	13.9 ^c^ ± 0.4	11.3 ^d^ ± 0.6	19.7 ^a^ ± 0.7	16.9 ^b^ ± 0.5
Protein content	31.8 ^a^ ± 0.2	24.3 ^c^ ± 0.4	26.7 ^b^ ± 1.1	26.8 ^b^ ± 0.3	28.3 ^b^ ± 0.4
Oil content	41.1 ^d^ ± 0.4	44.8 ^b^ ± 0.2	49.3 ^a^ ± 0.2	45.1 ^b^ ± 0.3	43.4 ^c^ ± 0.2

Means in the same row denoted by a different letter indicate significant difference between genotypes. *p* ≤ 0.05. No. = Number.

**Table 2:** Fatty acid composition of oil (%) from different accessions of *Brassica accessions*.

Compounds	Fay1	Fay2	Fay3	Fay4	Fay6
C14 Myristic	0.04 * ± 0.01	0.05 ± 0.01	0.06 ± 0.01	0.04 ± 0.01	0.04 ± 0.01
C16 Palmitic	3.16 ± 0.41	3.08 ± 0.40	3.28 ± 0.43	3.31 ± 0.43	3.37 ± 0.44
C16:1 Palmitoleic	0.08 ± 0.01	0.13 ± 0.02	0.22 ± 0.03	0.08 ± 0.01	0.12 ± 0.02
C17 Margaric	0.05 ± 0.01	0.06 ± 0.01	0.03 ± 0.00	0.07 ± 0.01	0.06 ± 0.01
C18 Stearic	0.88 ± 0.12	0.77 ± 0.10	1.12 ± 0.15	0.82 ± 0.11	0.93 ± 0.12
C18:1 n9 cis Oleic	7.73 ± 1.12	7.02 ± 0.92	22.98 ± 3.00	8.30 ± 1.08	9.90 ± 1.29
C18:1 n9 t Elaidic	0.11 ± 0.01	0.06 ± 0.01	0.03 ± 0.00	0.11 ± 0.01	0.08 ± 0.01
C18:2 n6 cis Linoleic	15.52 ± 2.03	15.65 ± 2.05	21.69 ± 2.84	16.44 ± 2.15	16.34 ± 2.14
C18:3 n3 Linolenic	14.14 ± 1.85	14.27 ± 1.87	14.02 ± 1.83	12.29 ± 1.61	12.11 ± 1.58
C20 Arachidic	0.73 ± 0.10	0.56 ± 0.07	0.71 ± 0.09	0.68 ± 0.09	0.74 ± 0.10
C20:1n11c Paullinic	6.45 ± 0.84	5.78 ± 0.76	11.01 ± 1.44	6.80 ± 0.89	6.54 ± 0.85
C20:1 n-9 Gondoic	0.97 ± 0.13	0.87 ± 0.11	0.81 ± 0.11	0.83 ± 0.11	0.80 ± 0.10
C22 Behenic acid	0.48 ± 0.06	0.49 ± 0.06	0.30 ± 0.04	0.47 ± 0.06	0.56 ± 0.07
C22 1 n9 Erucic	45.87 ± 6.0	47.78 ± 6.25	20.83 ± 2.72	45.88 ± 6.00	44.25 ± 5.78
C20 5n3 Eicosapentaenoic	1.23 ± 0.16	1.28 ± 0.17	0.29 ± 0.04	1.15 ± 0.15	1.12 ± 0.15
C22 4n6 Docosatetraenoic	1.67 ± 0.22	1.71 ± 0.22	0.91 ± 0.1	1.66 ± 0.22	1.98 ± 0.26
Unknown compound	0.89 ± 0.12	0.44 ± 0.06	1.71 ± 0.22	1.07 ± 0.14	1.06 ± 0.14
Total SFA	5.34 ± 0.70	5.01 ± 0.65	5.50 ± 0.72	5.39 ± 0.70	5.70 ± 0.75
Total unsaturated	93.77 ± 12.26	94.55 ± 12.36	92.79 ± 12.13	93.54 ± 12.23	93.24 ± 12.19
Total MUFA	61.21 ± 8.0	61.64 ± 8.08	55.88 ± 7.30	62.00 ± 8.10	61.69 ± 8.06
Total PUFA	32.56 ± 4.26	32.91 ± 4.03	36.91 ± 4.82	31.54 ± 4.12	31.55 ± 4.12
ω-6/ω-3	1.12 ± 0.15	1.12 ± 0.15	1.58 ± 0.21	1.35 ± 0.18	1.38 ± 0.18
PUFA/SFA	6.10 ± 0.80	6.57 ± 0.86	6.71 ± 0.88	5.85 ± 0.76	5.54 ± 0.72
SFA/UnSFA	0.06 ± 0.01	0.05 ± 0.01	0.06 ± 0.01	0.06 ± 0.01	0.06 ± 0.01
SFA/PUFA	0.16 ± 0.02	0.15 ± 0.02	0.15 ± 0.02	0.17 ± 0.02	0.18 ± 0.02
Oleic/linoleic	0.50 ± 0.07	0.45 ± 0.06	1.06 ± 0.14	0.50 ± 0.07	0.61 ± 0.08

* Results are expressed as percentage of the total fatty acids. SFA: saturated fatty acids, MUFA: monounsaturated fatty acids, PUFA: polyunsaturated fatty acids, UnSFA: unsaturated fatty acids.

**Table 3:** Direct and indirect effects, pearson correlation and coefficient of determination (R^2^) of different agronomic traits and quality characteristics on oil content (%) of five genotypes of *Brassica* spp.

Characters	Direct Effect	Indirect Effect Via								
		PH	HFB	NPB	NPP	SYP	Protein	PL	NSP	TIE
PH	−0.16		0.82	−0.08	−0.62	−0.32	0.13	−0.11	−0.11	−0.29
HFB	−1.95	0.07		0.19	1.58	0.42	−0.11	0.31	0.27	2.73
NPB	−0.20	−0.07	1.87		−1.54	−0.41	0.12	−0.31	−0.26	−0.6
NPP	−0.19	−0.06	1.92	−1.61		−0.41	0.10	−0.31	−0.27	−0.64
SYP	0.48	−0.11	1.69	−0.17	−1.36		0.15	−0.25	−0.23	−0.28
Protein	−0.23	−0.10	0.97	−0.10	−0.73	−0.32		−0.18	−0.14	−0.6
PL	−0.33	−0.06	1.84	−0.18	−1.50	−0.37	0.13		−0.26	−0.4
NSP	−0.28	−0.06	1.90	−0.18	−1.56	−0.40	0.11	−0.30		−0.49
Person correlation with oil percent (r)		−0.46	0.77	−0.79	−0.82	−0.76	−0.37	−0.74	−0.78	
coefficient of determination (R^2^)		21.16%	59.29%	62.41%	67.24%	57.76%	13.69%	54.76%	60.84%	
*p*−value		0.00 **	0.00 **	0.00 **	0.00 **	0.00 **	0.04 *	0.00 **	0.00 **	

Note: Total indirect effect (TIE), plant height (PH), height to first branches (HFB), number of pods per plant (NPP), seed yield per plant (SYP), protein (%), pods length (PL), number of primary branches (NPB), simple linear correlation (r), coefficient of determination (R^2^) and **, *p* < 0.01; *, *p* < 0.05.

## Data Availability

The data presented in this study are available upon request from the corresponding author.

## Abbreviations

Internal Transcribed Spacer (ITS); Restriction Fragment Length Polymorphism (RFLP); Polymerase Chain Reaction (PCR); Plant Height (PH); Height of First Branch (HFB); Number of Primary Branches/Plant (NPB); Number of Pods/Plant (NPP); Pods Length (PL); Number of Seeds/Pods (NSP); Seed yield/plant (SYP); Adenin (A); Guanin (G); Cytosin (C); Thymin (T); Coronary Heart Disease (CHD); Low-Density Lipoprotein (LDL); World Health Organization (WHO); Saturated Fatty Acids (SFA); Unsaturated Fatty Acids (UnSFA); Monounsaturated Fatty Acids (MUFAs); Polyunsaturated Fatty Acids (PUFAs).

## References

[1] Balkaya A., Yanmaz R., Apaydin A., Kar H. (2005). Morphological characterisation of white head cabbage (*Brassica oleracea* var. capitata subvar. alba) genotypes in Turkey. N. Z. J. Crop Hortic. Sci..

[2] El-esawi M.A., Bourke P., Germaine K., Malone R. (2012). Assessment of Morphological Variation in Irish *Brassica oleracea* Species. J. Agric. Sci..

[3] Kİbar B., Karaağaç O., Kar H. (2016). Determination of Morphological Variability among Cabbage (*Brassica oleracea* var. capitata L.) Hybrids and Their Parents. J. Inst. Sci. Technol..

[4] Seifert K.A., Samson R.A., Dewaard J.R., Houbraken J., Lévesque C.A., Moncalvo J.-M., Louis-Seize G., Hebert P. (2007). Prospects for fungus identification using CO1 DNA barcodes, with Penicillium as a test case. Proc. Natl. Acad. Sci. USA.

[5] Cardone M., Mazzoncini M., Menini S., Rocco V., Senatore A., Seggiani M., Vitolo S. (2003). *Brassica carinata* as an alternative oil crop for the production of biodiesel in Italy: Agronomic evaluation, fuel production by transesterification and characterization. Biomass Bioenergy.

[6] Thakur A.K., Singh K.H., Sharma D., Parmar N., Nanjundan J. (2019). Breeding and genomics interventions in Ethiopian mustard (*Brassica carinata A. Braun*) improvement—A mini review. S. Afr. J. Bot..

[7] Hagos R., Shaibu A.S., Zhang L., Cai X., Liang J., Wu J., Lin R., Wang X. (2020). Ethiopian Mustard (*Brassica carinata A. Braun*) as an Alternative Energy Source and Sustainable Crop. Sustainability.

[8] Ali W.M., Pant D.P. (2013). Genetic analysis of oil content in Ethiopian mustard (*Brassica carinata* A. Braun). Pantnagar J. Res..

[9] Martínez-Valdivieso D., Font R., Del Río-Celestino M. (2019). Prediction of agro-morphological and nutritional traits in Ethiopian mustard leaves (*Brassica carinata* A. Braun) by visible-near-infrared spectroscopy. Foods.

[10] Tiwari M., Babu V.B., Kotwaliwale N., Hamad R., Singh K. (2017). Identification of Indian mustard (*Brassica juncea* L.) varieties by DUS testing using morphological characters Identification of Indian mustard (*Brassica juncea* L.) varieties by DUS testing using morphological characters. J. Oilseed Brassica.

[11] Weerakoon S.R., Iqbal M.C.M., Somaratne S., Peiris P.K.D., Wimalasuriya W.S.R. Delimitation of local mustard (*Brassica juncea*) germplasm in Sri Lanka and improvement of their nutritive quality. Proceedings of the 12th International Rapeseed Congress.

[12] Weerakoon S., Somaratne S. (2010). Agro-morphological characterization and relationships amoung mustard germplasm (*Brassica juncea* [ L.] Czern & Coss ) in Sri Lanka: A classification tree approach. J. Agric. Sci..

[13] Timpano E.K., Scheible M.K.R., Meiklejohn K.A. (2020). Optimization of the second internal transcribed spacer (*ITS2*) for characterizing land plants from soil. PLoS ONE.

[14] Ziffer-berger J., Keren-keiserman A., Doron-faigenboim A. (2019). Notes on the generic position of *Brassica deserti* (Brassicaceae). Isr. J. Plant Sci..

[15] Takamiya T., Wongsawad P., Tajima N., Shioda N., Lu A.J.F., Wen C.L., Bin Wu J., Handa T., Iijima H., Kitanaka S. (2011). Identification of Dendrobium Species Used for Herbal Medicines Based on Ribosomal DNA Internal Transcribed Spacer Sequence. Biol. Pharm. Bull..

[16] Panyal N., Panwar S., Sonah H., Singh K.P., Sharma T.R. (2013). Genetic diversity analysis of marigold (Tagetes sp) genotypes using RAPD and ISSR markers. Indian J. Agric. Sci..

[17] Lee S., Mohamed R., Faridah-Hanum I., Lamasudin D. (2018). Utilization of the internal transcribed spacer (ITS) DNA sequence to trace the geographical sources of *Aquilaria malaccensis* Lam. populations. Plant Genet. Resour..

[18] Rush T.A., Golan J., McTaggart A., Kane C., Schneider R.W., Aime M.C. (2019). Variation in the Internal Transcribed Spacer Region of *Phakopsora pachyrhizi* and Implications for Molecular Diagnostic Assays. Plant Dis..

[19] Bhandari H.R., Bhanu A.N., Srivastava K., Singh M.N., Hemantaranjan A. (2017). Assessment of genetic diversity in crop plants—An overview. Adv. Plants Agric. Res..

[20] Zeng X., Li H., Li K., Yuan R., Zhao S., Li J., Luo J., Li X., Ma H., Wu G. (2021). Evolution of the Brassicaceae-specific MS5-Like family and neofunctionalization of the novel MALE STERILITY 5 gene essential for male fertility in *Brassica napus*. New Phytol..

[21] Crevelari J.A., Durães N.N.L., Bendia L.C.R., Vettorazzi J.C.F., Entringer G.C., Junior J.A.F., Pereira M.G. (2018). Correlations between agronomic traits and path analysis for silage production in maize hybrids. Bragantia.

[22] Granlund M., Zimmerman D.C. (1975). Oil content of safflower seeds as determined by wide-line nuclear magnetic resonance (NMR). Proc. Natl. Acad. Sci. USA.

[23] Velioglu S.D., Temiz H.T., Ercioglu E., Velioglu H.M., Topcu A., Boyaci I.H. (2017). Use of Raman spectroscopy for determining erucic acid content in canola oil. Food Chem..

[24] Mohdaly A.A., Mahmoud A.A., Housain M.H., Iryna S. (2015). Chemical composition, physicochemical properties and fatty acid profile of Tiger Nut (*Cyperus esculentus* L) seed oil as affected by different preparation methods. Int. Food Res. J..

[25] Abdi H., Salkind N.J. (2007). Bonferroni and Sidak Corrections for Mutliple Comparisons.

[26] Dewey D.R., Lu K.H. (1959). A correlation and path coefficient analysis of components of crested wheat grass seed production. Agron. J..

[27] Arbuckle J.L. (2017). IBM SPSS Amos 24 User’s Guide.

[28] Doyle J.J., Doyle J.L. (1990). Isolation of plant DNA from fresh tissue. Focus.

[29] AbdAl-Aziz S.A., El-Metwally M.M., Saber W.I. (2012). Molecular identification of a novel inulinolytic fungus isolated from and grown on tubers of *Helianthus tuberosus* and statistical screening of medium components. World J. Microbiol. Biotechnol..

[30] Hewavitharana G.G., Perera D.N., Navaratne S.B., Wickramasinghe I. (2020). Extraction methods of fat from food samples and preparation of fatty acid methyl esters for gas Chromatography: A Review. Arab. J. Chem..

[31] Froyen E., Burns-Whitmore B. (2020). The Effects of Linoleic Acid Consumption on Lipid Risk Markers for Cardiovascular Disease in Healthy Individuals: A Review of Human Intervention Trials. Nutrients.

[32] Babaoglu M., Guel M. (2015). Safflower (*Carthamus tinctorius* L.) breeding activities at Trakya Agricultural. J. Crop Breed. Genet..

[33] Kose A., Onder O., Bilir O., Kosar F. (2018). Application of multivariate statistical analysis for breeding strategies of spring safflower (*Carthamus tinctorius* L.). Turkish J. Field Crops.

[34] Teklehaymanot T., Wang H., Liang J., Wu J., Lin R., Zhou Z., Cai X., Wang X. (2019). Variation in Plant Morphology and Sinigrin Content in Ethiopian Mustard (*Brassica carinata* L.). Hortic. Plant J..

[35] Hossain Z., Johnson E.N., Wang L., Blackshaw E.R., Gan Y. (2019). Comparative analysis of oil and protein content and seed yield of five Brassicaceae oilseeds on the Canadian prairie. Ind. Crop Prod..

[36] Stamigna C., Chiaretti D., Chiaretti E., Prosini P.P. (2012). Oil and furfural recovery from *Brassica carinata*. Biomass Bioenerg..

[37] Aghdam A.M., Sayfzadeh S., Shirani Rad A.H., Valadabadi S.A., Zakerin H.R. (2019). The assessment of water stress and delay cropping on quantitative and qualitative traits of rapeseed genotypes. Ind. Crop Prod..

[38] Cartea E., De Haro-Bailón A., Padilla G., Obregón-Cano S., Del Rio-Celestino M., Ordás A. (2019). Seed Oil Quality of *Brassica napus* and *Brassica rapa* Germplasm from Northwestern Spain. Foods.

[39] Zahran H.A., Abd-Elsaber A., Tawfeuk H.Z. (2020). Genetic diversity, chemical composition and oil characteristics of six sesame genotypes. OCL.

[40] Kumar S., Seepaul R., Mulvaney M.J., Colvin B., George S., Marois J.J., Bennett R., Leon R., Wright D.L., Small I.M. (2020). *Brassica carinata* genotypes demonstrate potential as a winter biofuel crop in South East United States. Ind. Crop Prod..

[41] Sokoła-Wysoczańska E., Wysoczański T., Wagner J., Czyż K., Bodkowski R., Lochyński S., Patkowska-Sokoła B. (2018). Polyunsaturated fatty acids and their potential therapeutic role in cardiovascular system disorders—A review. Nutrients.

[42] Kirkegaard J.A., Lilley J.M., Brill R.D., Ware A.H., Walela C.K. (2018). The critical period for yield and quality determination in canola (*Brassica napus* L.). Field Crop. Res..

[43] Abebe D. (2006). Genetic Variability and Association among Seed Yield and Yield Related Traits in Ethiopian Mustard (*Brassica carinata* A. Braun) at Kulumsa, Arsi. Master’s Thesis.

[44] Rondanini D.P., Vilarino M.d.P., Roberts M.E., Polosa M.A., Botto J. (2014). Physiological responses of spring rapeseed (*Brassica napus*) to red/far-red ratios and irradiance during pre- and post-flowering stages. Physiol. Plant..

[45] Paul M., Panda G., Kumar P., Mohapatra D., Thatoi H. (2020). Study of structural and molecular interaction for the catalytic activity of cellulases: An insight in cellulose hydrolysis for higher bioethanol yield. J. Mol. Struct..

[46] Ton L.B., Neik T.X., Batley J. (2020). The Use of Genetic and Gene Technologies in Shaping Modern Rapeseed Cultivars (*Brassica napus* L.). Genes.

[47] Zuppa A., Costantini S., Costantini M. (2014). Comparative sequence analysis of bacterial symbionts from the marine sponges *Geodia cydonium* and *Ircinia muscarum*. Bioinformation.

[48] Kaya I., Demir I., Usta M., Sipahioglu M.H. (2020). Molecular phylogeny based on its sequences of nrDNA of some species belonging to dodder (*Cuscuta* L.) genus from various ecological sites of Turkey. Not. Bot. Horti Agrobot Cluj-Napoca.

[49] Elshafei A.A., Afiah S.A., Amer M.A., El-enany M.A.M. (2019). Validation of molecular markers linked with salinity tolerance in wheat (*Triticum aestivum* L.) grown on saline soil. Biosci. Res..

[50] Paul M., Islam T., Hoque M.I., Sarker R.H. (2020). Analysis of genetic diversity in oilseed brassica germplasm through issr markers and isozyme profiling. Bangladesh J. Bot..

